# MicroRNA-148a regulates low-density lipoprotein metabolism by repressing the (pro)renin receptor 

**DOI:** 10.1371/journal.pone.0225356

**Published:** 2020-05-21

**Authors:** Na Wang, Lishu He, Hui Lin, Lunbo Tan, Yuan Sun, Xiaoying Zhang, A. H. Jan Danser, Hong S. Lu, Yongcheng He, Xifeng Lu

**Affiliations:** 1 Department of Physiology, Shenzhen University Health Science Center, Shenzhen University, Shenzhen, China; 2 Division of Pharmacology and Vascular Medicine, Department of Internal Medicine, Erasmus Medical Center, Rotterdam University, Rotterdam, The Netherlands; 3 Department of Pharmacology and Toxicology, Medical College of Wisconsin, Milwaukee, Wisconsin, United States of America; 4 Translational Medicine Collaborative Innovation Center, The Second Clinical Medical College (Shenzhen People’s Hospital) of Jinan University, Shenzhen, China; 5 Saha Cardiovascular Research Center and Department of Physiology, University of Kentucky, Lexington, Kentucky, United States of America; 6 Department of Nephrology, Shenzhen Hengsheng Hospital, Shenzhen, China; Beijing Key Laboratory of Diabetes Prevention and Research, CHINA

## Abstract

High plasma LDL cholesterol (LDL-c) concentration is a major risk factor for atherosclerosis. Hepatic LDL receptor (LDLR) regulates LDL metabolism, and thereby plasma LDL-c concentration. Recently, we have identified the (pro)renin receptor [(P)RR] as a novel regulator of LDL metabolism, which regulates LDLR degradation and hence its protein abundance and activity. *In silico* analysis suggests that the (P)RR is a target of miR-148a. In this study we determined whether miR-148a could regulate LDL metabolism by regulating *(P)RR* expression in HepG2 and Huh7 cells. We found that miR-148a suppressed *(P)RR* expression by binding to the 3’-untranslated regions (3’-UTR) of the (P)RR mRNA. Mutating the binding sites for miR-148a in the 3’-UTR of (P)RR mRNA completely abolished the inhibitory effects of miR-148a on *(P)RR* expression. In line with our recent findings, reduced *(P)RR* expression resulted in decreased cellular LDL uptake, likely as a consequence of decreased LDLR protein abundance. Overexpressing the (P)RR prevented miR-148a-induced reduction in LDLR abundance and cellular LDL uptake. Our study supports a new concept that miR-148a is a regulator of *(P)RR* expression. By reducing (P)RR abundance, miR-148a decreases LDLR protein abundance and consequently cellular LDL uptake.

## Introduction

Elevated plasma LDL cholesterol (LDL-c) level is a major risk factor for cardiovascular diseases (CVD), a leading cause of world-wide death. Plasma LDL is mainly cleared by the LDLR in the liver. Genetic mutations resulting in defective LDLR functions are associated with elevated plasma LDL-c levels and increased risks for CVD [1]. Additionally, decreased LDLR protein abundance, caused either by reduced transcription or increased protein degradation, leads to disturbed LDL clearance. Due to its importance in regulating LDL metabolism, hepatic LDLR transcription is tightly controlled under normal physiological state by sterol regulatory element-binding protein in response to cellular cholesterol levels [2]. Protein degradational regulation is also important for controlling LDLR abundance. Several factors, such as protein convertase subtilisin/kexin9 (PCSK9) and inducible degrader of LDLR (IDOL), have been found to promote LDLR degradation [3–5]. Dysregulation of these regulatory factors, caused either by genetic mutations or pathological conditions, is associated with altered plasma LDL levels and CVD risks [6, 7].

Recently, we have identified the (pro)renin receptor [(P)RR] as a novel regulator of LDLR [8]. The (P)RR can bind renin, and prorenin with higher affinity, playing an important role in local activation of the renin-angiotensin system (RAS). Upon renin/prorenin binding, the (P)RR triggers intracellular signaling cascades, such as signal-regulated kinase 1/2 and p38 kinase signaling, resulting in upregulation of profibrotic factors including transforming growth factor β, collagen-1 and fibronectin [9–11]. The (P)RR has also been reported to play crucial roles in maintaining vacuolar H^+^-ATPase integrity, Wnt/β-catenin and PCP signaling, glucose metabolism, and lipid metabolism, independent of renin/prorenin [12–16]. Despite its importance in several physiological processes, it is unclear how the (P)RR itself is being regulated. Interestingly, renin/prorenin-(P)RR signaling suppresses (P)RR expression via a negative feedback mechanism [17]. Altered (P)RR expression has been reported in several tissues under pathophysiological conditions, such as in the adipose tissue of HFD-fed obese mice and in the kidney of diabetic nephropathy (DN) mice [18], and may play a role in the onset and development of such diseases.

Recent studies have found that several microRNAs (miRNAs) regulate (P)RR expression, suggesting that miRNAs may play a role in regulating (P)RR functions [19]. MiRNAs are short non-coding RNAs about 22 nucleotides in length, which can affect protein abundance of target genes via non-sense mediated messenger RNA decay or translational inhibition by binding to the 3’-untraslated regions (3’-UTR) of the mRNA of target gene [20]. With their ability to regulate gene expression, miRNAs have been reported to play a role in a wide variety of pathophysiological processes, such as hypertension, obesity, and atherosclerosis [21–23]. Using *in silico* analysis, we found that (P)RR is a potential target of miR-148a. Moreover, increased circulating miR-148a levels are associated with elevated plasma LDL-c levels, linking miR-148a with plasma cholesterol homeostasis regulation [24]. As we recently discovered, inhibiting the (P)RR reduces LDLR protein abundance and thus affecting LDL metabolism both *in vitro* and *in vivo* [8, 16]. We hypothesized that down-regulation of the (P)RR and its consequent effects on LDLR could be a mechanism explaining how miR-148a affects LDL metabolism.

To test this hypothesis, we first determined whether miR-148a affects *(P)RR* expression, and its consequences on cellular LDL uptake in human hepatic cells. We found that miR-148a potently reduced *(P)RR* expression and cellular LDL uptake in HepG2 and Huh7 cells. Blocking miR-148a using its inhibitors attenuated miR-148a-induced reduction in *(P)RR* expression and cellular LDL uptake. Furthermore, we found that overexpressing the (P)RR prevented miR-148a-induced reduction in cellular LDL uptake. Our study thus shows that miR-148a regulates LDL metabolism via (P)RR-LDLR axis.

## Materials and methods

### Chemicals and reagents

All chemicals and reagents were purchased from Sigma Aldrich with highest purity unless where else indicated.

### Cell culture and transfection

HepG2, Huh7 and HEK293 cells were maintained in DMEM GlutaMAX (Thermo Fisher Scientific) supplemented with 10% fetal bovine serum (Thermo Fisher Scientific), 100 U/mL penicillin and 100 mg/mL streptomycin (Thermo Fisher Scientific) at 37°C and 5% CO_2_. Unless when measuring LDL uptake (quantitatively or microscopically), all cellular experiments were performed with cells cultured with normal culture medium containing serum. For miRNA mimics transfection in HEK293, HepG2 and Huh7 cells, Lipofectamine RNAimax (Thermo Fisher Scientific) was used following the manufacturer’s protocol. MiRNA mimics and inhibitors were synthesized by GenePharm (Suzhou, China) and their sequences were listed in **S1 Table**. For plasmid transfection, Lipofectamine 2000 (Thermo Fisher Scientific) was used for transfecting HEK293 cells, and Lipofectamine 3000 (Thermo Fisher Scientific) was used for transfecting HepG2 and Huh7 cells.

### Immunoblotting

For immunoblotting, cells were washed twice with ice-cold PBS and lysed at 4°C in RIPA buffer (containing 50 mmol/L Tris-HCl, 150 mmol/L NaCl, 1% Triton X-100, 0.1% SDS, 1% sodium deoxycholate, and complete protease inhibitor; pH 7.4). Lysates were centrifuged at 10,000 g for 10 minutes at 4°C, and protein concentrations in the supernatant were measured with a bicinchoninic acid assay kit (Thermo Fisher Scientific). Equal amount of proteins (20–30 μg) were resolved by 4–20% Bis-Tris gels (GeneScript) and transferred to PVDF membranes using iBlot^®^ 2 Dry Blotting System (Thermo Fisher Scientific). Blots were probed using previously described primary antibodies: anti-LDLR (1:1000; Proteintech), anti-(P)RR (1:1000), and anti-tubulin (1:5000; Proteintech), and HRP-conjugated goat anti-mouse or goat anti-rabbit antibodies (1:5000; Jackson ImmunoResearch) and detected by ECL. Abundance of target protein was quantified by measuring band intensity using Image J, and corrected for the band intensity of β-tubulin of the same sample.

### RNA isolation and qPCR

Total RNA was isolated from cells using TRIzol (Thermo Fisher Scientific) and Direct-zol^TM^ RNA MiniPrep kit (ZYMO Research) following the manufacturer’s protocol. One microgram of total RNA was reverse transcribed with Prime Script^TM^ RT Master Mix (TaKaRa). Quantitative PCR assays were performed on a qTOWER apparatus (Analytic Jena) using SYBR Green master mix (TaKaRa). Gene expression was normalized for the expression of 36B4, and expressed as mean ± SEM. Sequences of the primers used in this study are provided in **S1 Table**.

### LDL uptake assay

Blood was drawn from healthy volunteer, and LDL was prepared by ultracentrifugation as previously described [25]. LDL was then labeled with DyLight-488 (Thermo Fisher Scientific) following manufacturer’s protocol, and LDL uptake was measured using DyLight-488-labeled LDL as previously described [26]. In brief, HepG2 and Huh7 cells were cultured for 16 hours prior to adding LDL in sterol-depleted medium [(DMEM supplemented with 10% bovine lipid-deficient serum (LPDS), 5 μg/mL simvastatin (Selleck), and 100 μM mevalonic acid (Sigma Aldrich)] as previously described [5]. Cells were then incubated with 5 μg/mL DyLight-488-labeled LDL in DMEM containing 0.5% BSA for 3 hours at 37°C or 4°C. In these experiments, 100 μg/mL non-labeled LDL was used to correct for non-specific LDL binding. After incubation, cells were washed twice by ice-cold PBS supplemented with 0.5% BSA and then lysed in RIPA buffer. Specific LDL uptake was calculated as the fluorescent intensity differences between 37°C and 4°C, after subtracting non-specific association/binding. LDL uptake was determined by quantification of the fluorescence signal using a multi-mode fluorescent microplate reader (Cytation 5, Biotek), and corrected for the protein contents in the same lysates as determined with the BCA assay. To visualize LDL uptake, cells were cultured on coverslips and treated as above mentioned, then fixed with 4% paraformaldehyde, and mounted with Vectorshield containing DAPI (Vector Laboratories). Prepared slides were visualized with Cytation 5 using slide imaging mode with a 40x objective lens.

### 3'-UTR luciferase reporter assay

Full length 3'-UTR of human (P)RR and LDLR were cloned into the luciferase reporter vector (Promega) using the following primers: 5’-CAGAATTCATGTTACCTGTGCCAGA-ATTAG-3’ (forward) and 5’- CACCTCGAGTCTATAGAATGAAGTTGTACCAC-3’ (reverse) for (P)RR, and 5’-CAGAATTCACATC-TGCCTGGAGTCCCGTCCC-3’ (forward) and 5’-CACCTCGAGAGACAAATTGGTTCATTTAAGAA-3’ (reverse) for LDLR. Mutant 3’-UTR luciferase reporter vectors were generated by PCR using site-directed mutagenesis technique. Correctness of the constructs were then confirmed by sequencing. HEK293 were transfected with constructed vectors, together with miR-148a mimics or control oligos. After 48 hours of transfection, cells were lysed using lysis buffer provided by Dual-Glo Luciferase Assay kit, and lysates were cleared as described above. Luciferase activities were measured using Dual-Glo Luciferase Assay (Promega). In short, *Firefly* luciferase activities in the lysates were measured, and corrected for *Renilla* luciferase activities in the same lysates.

### Statistical analysis

Data are presented as mean ± SEM. Data sets were first analyzed using D’Agostino-Pearson omnibus test for normality. F test, Browne-Forsythe test or Bartlett’s test was performed for testing if the variances of different data sets are equal. When passing normality and equal variance test, parametric Student’s t-test was performed for comparison of two groups, or One-way ANOVA followed by the Bonferroni correction was performed for comparison of more than two groups. When failed passing normality and equal variance test, non-parametric Student’s t-test with Welch’s correction was performed, or non-parametric One-Way ANOWA followed by Dunn’s correction was performed for comparison of more than two groups. P values of <0.05 were considered significant. Statistical analysis was performed using Prism 7 (Graphpad Software).

## Results

### MiR-148a reduced (P)RR expression and protein abundance

**Fig 1:**
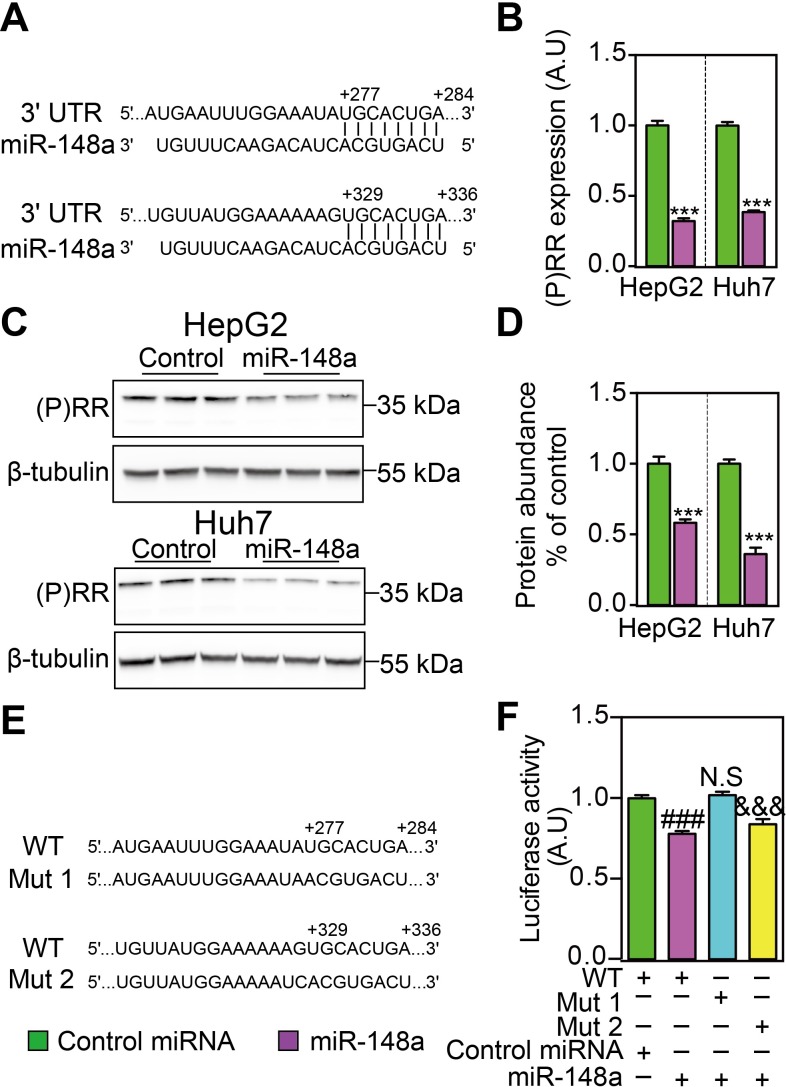
MiR-148a regulates (P)RR expression by targeting its 3’-UTR sequences. **A**. An illustration showing that there are two predicted binding sites of miR-148a on the 3’-UTR of human (P)RR. HepG2 and Huh7 cells, cultured with serum containing medium, were transfected with miR-148a or control miRNA for 48 hours, and gene expression and protein abundances were analyzed. **B.** *(P)RR* mRNA level was determined by quantitative PCR, and corrected for 36B4 level in the same sample and expressed as ratio of the control miRNA transfected. Results are from four independent experiments in triplicates. N = 12; ***: p<0.001. **C**. Total cell lysates were blotted as indicated and a representative blot of 3 independent experiments in triplicates was shown. **D**. (P)RR protein abundance was quantified and normalized to the level of tubulin in the same lysates, and expressed as the relative ratio of (P)RR abundance in control miRNA transfected. N = 9; ***: P<0.001. **E.** An illustration showing two constructs (Mut1 and Mut2) which are mutated for the binding site for miR-148a on the 3’-UTR of human (P)RR, comparing to wildtype (WT) sequence. **F.** HEK293T cells were transfected with luciferase reporter plasmids constructed using wildtype (WT) and mutated (Mut1 and Mut2) 3’-UTR of human (P)RR, together with either control miRNA or miR-148a. Cells were cultured with serum containing medium. Firefly luciferase activity was measured and corrected for Renilla luciferase activity in the same sample, and expressed as ratio of WT reporter plasmid transfected samples. Results are from four independent experiments in triplicates (N = 12). ###: WT+miR-148a vs. WT+control miRNA, p<0.001; N.S (not significant): Mut1+miR-148a vs. WT+miR-148a; &&&: Mut2+miR-148a vs. WT, p<0.001.

Using TargetScan (http://www.targetscan.org/), we performed *in silico* analysis and found that there were two putative binding sites for miR-148a in the 3’-UTR of the (P)RR (**Fig 1A**). We thus asked whether miR-148a could regulate (P)RR expression and functions. Firstly, we tested the effects of miR-148a on *(P)RR* expression in human hepatic cells. Transfecting HepG2 and Huh7 cells with miR-148a mimics effectively reduced (P)RR transcript levels by ~60–70% (**Fig 1B**), and consequently (P)RR protein abundance by 40–60% (**Fig 1C and 1D**). To confirm whether miR-148a directly regulates *(P)RR* expression, we mutated the putative bindings sites in the 3’-UTR of (P)RR (**Fig 1E**), and studied their consequences on gene expression using luciferase assay. Mutating the binding site 1, but not the binding site 2, attenuated miR-148a-induced reduction in luciferase activity (**Fig 1F**), confirming that the (P)RR is a direct target of miR-148a.

### MiR-148a reduced LDLR abundance and cellular LDL uptake

**Fig 2:**
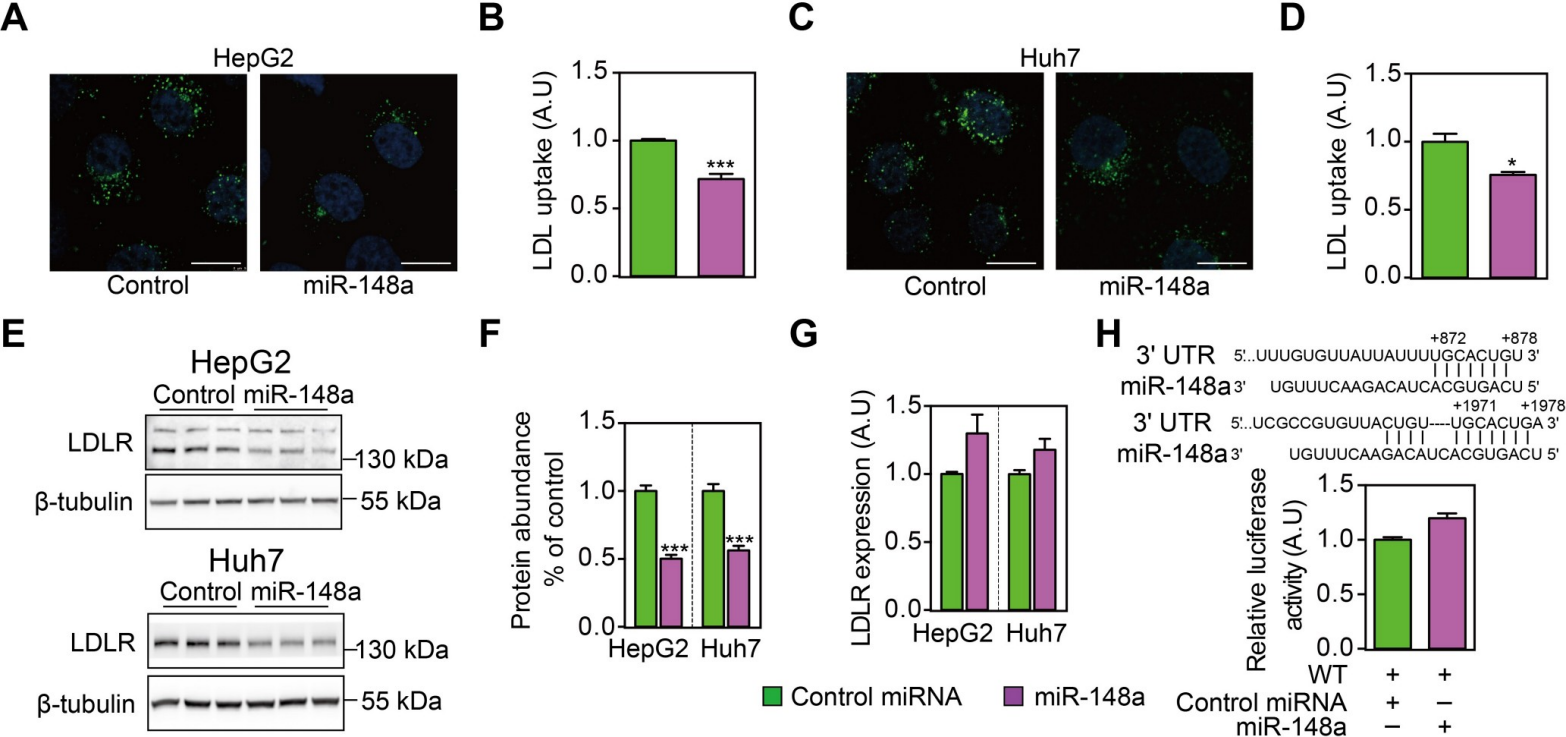
MiR-148a reduces cellular LDL uptake by reducing LDLR protein abundance but not its transcript levels. HepG2 (**A & B**) and Huh7 cells (**C & D**) were transfected with control miRNA or miR-148a for 32h, and then treated with LSM for 16h before being incubated with 5 μg/mL Dylight-488-labeled LDL for 3h. **A & C**. Representative fluorescence images of cells. Nuclei are counterstained with 4’6-diaminido-2-phenylindole (blue). Scale bar: 10 μm. **B & D**. Quantitative measurement of LDL uptake in cells. Results are from four independent experiments in triplicates. N = 12; *: p<0.01; ***: p<0.001. **E**. HepG2 and Huh7 cells were transfected with control miRNA or miR-148a for 48 hours, and cultured under serum-containing medium. Total cell lysates were immunoblotted as indicated, and representative blots of 3 independent experiments in triplicates were shown. **F**. LDLR protein abundance was quantified and normalized to the level of tubulin in the same lysates, and expressed as the relative ratio of LDLR abundance in control miRNA transfected. N = 9. ***: p<0.001. **G**. HepG2 and Huh7 cells were transfected as above and culture under serum-containing medium, and *LDLR* mRNA levels were determined by quantitative PCR. Results are from four independent experiments in triplicates. N = 12; *: p<0.05. **H**. Putative miR-148a binding sites were identified in the 3’-UTR of human LDLR, and luciferase reporter plasmid was constructed accordingly. HEK293T cells were transfected the reporter plasmid, and cultured under serum-containing medium. Firefly luciferase activity was measured and corrected for Renilla luciferase activity in the same sample, and expressed as ratio of WT 3’-UTR transfected samples. Results are from four independent experiments in triplicates (N = 12).

Next, we tested if miR-148a could affect cellular LDL uptake as a consequence of reduced (P)RR abundance. As expected, miR-148a transfection effectively reduced cellular LDL uptake in HepG2 and Huh7 cells (**Fig 2A–2D**). A previous study shows that miR-148a could suppress *LDLR* expression in hepatic cells[24]. Thus, it is possible that miR-148a directly down-regulates *LDLR*, causing observed phenotype. In line with this, we found that miR-148a reduced LDLR protein abundance in HepG2 and Huh7 cells (**Fig 2E and 2F**). However, LDLR transcript levels were not reduced but tends to be increased by miR-148a transfection (**Fig 2G**), which likely reflects reduced intracellular cholesterol levels as LDLR functions is limited. To further validate this observation, we generated a luciferase reporter vector by cloning the full length 3’-UTR of LDLR, and tested the effect of miR-148a on luciferase activity. Despite the presence of two putative binding sites (one conserved and one non-conserved) in the 3’-UTR of LDLR, miR-148a failed to reduce luciferase activity (**Fig 2H**). Moreover, transfecting the cells with siRNA targeting the 3’-UTR of LDLR potently reduced luciferase activity, validated the correctness of the reporter vector constructed (**S1 Fig**). As these experiments were performed in cells cultured with normal medium containing serum rather than LPDS, we further assessed the effect of miR-148a on LDLR and ABCA1 expression in HepG2 cells treated with LPDS and LSM. Yet, miR-148a was still unable to reduce LDLR and ABCA1 transcript levels under cholesterol-depleted conditions (**S2 Fig**). Taken together, our results suggest that miR-148a reduces cellular LDL uptake by decreasing LDLR protein abundance, without affecting its transcript levels.

### MiR-148a regulated LDL uptake via modulating (P)RR levels

**Fig 3:**
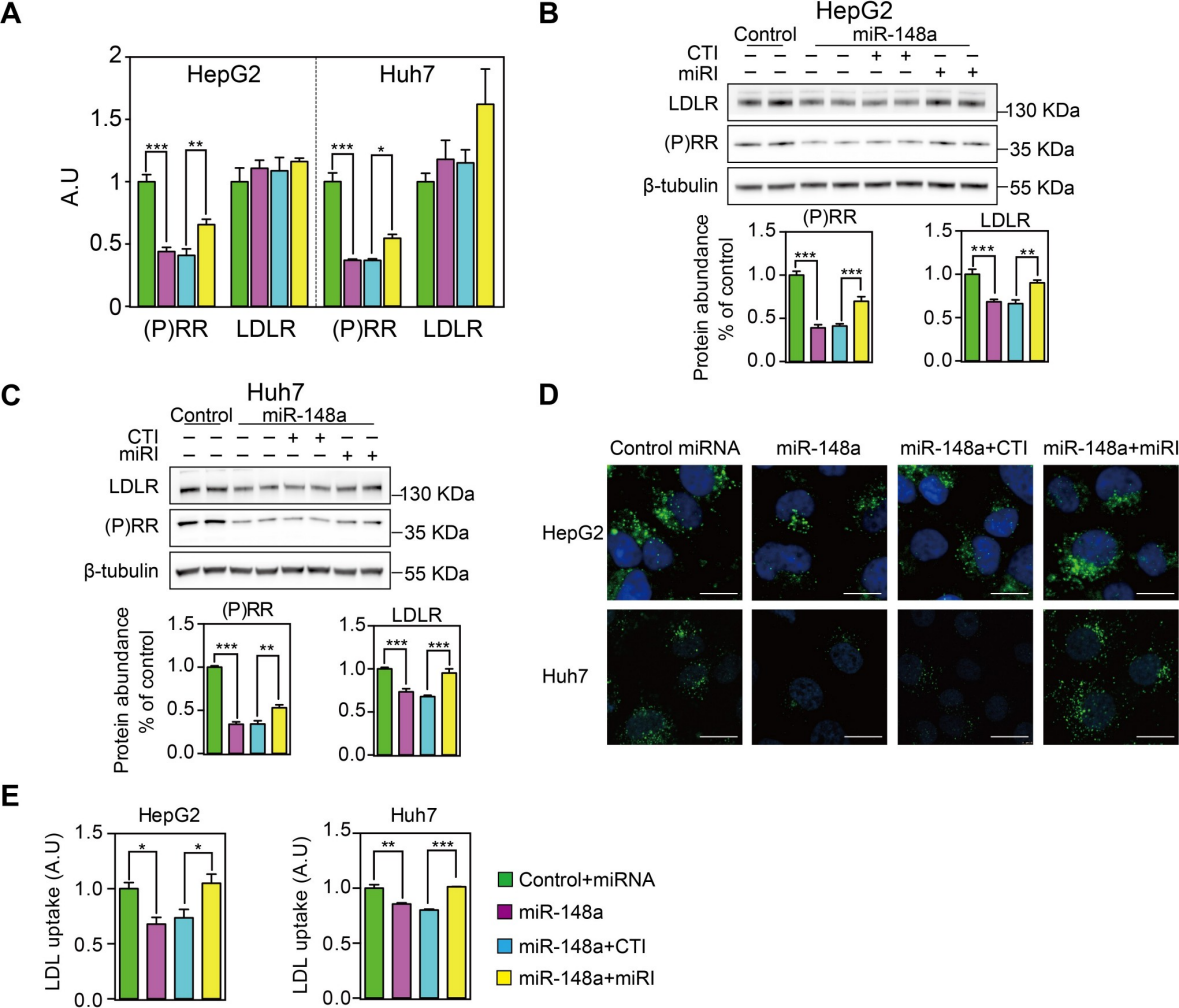
MiR-148a reduces cellular LDL uptake by specifically modulating *(P)RR* expression. HepG2 and Huh7 cells were transfected with control miRNA or miR-148a, together with control inhibitor (CTI) or miR-148a inhibitor (miRI) for 48 hours. Cells were cultured with serum containing medium. **A**. (P)RR and LDLR mRNA levels were determined by quantitative PCR. Results are from 4 independent experiments in triplicates. N = 12; **: p<0.01; ***: p<0.001. **B & C.** HepG2 (**B**) and Huh7 cells (**C**) were treated as above, and total cell lysates were immunoblotted as indicated. Representative blots of 4 independent experiments in duplicate were shown. (P)RR and LDLR protein abundance were quantified. N = 8. **: p<0.01; ***: p<0.001. **D & E**. HepG2 and Huh7 cells were treated as above, and medium were changed to LSM 16 prior to incubation with 5 μg/mL Dylight-488-labeled LDL for 3 hours. **D**. Representative fluorescence images. Scale bar: 10 μm. **E**. Quantitative measurement of LDL uptake. Results are from 3 independent experiments in triplicates. N = 9; *: p<0.01; ***: p<0.001.

To explore whether miR-148a regulates LDL metabolism specifically by controlling (P)RR levels, we first tested the effects of miR-148a inhibitors on (P)RR levels and cellular LDL uptake. As expected, transfecting cells with miR-148a inhibitors, but not control inhibitors, attenuated miR-148a-induced reduction in *(P)RR* transcript levels in HepG2 and Huh7 cells (**Fig 3A**). As expected, incubating cells with miR-148a or together with its inhibitors did not affect *LDLR* expression, further supporting that LDLR is not a direct target of miR-148a, at least not in the hepatic cells tested. Similar to the changes in mRNA, blocking miR-148a with its inhibitors reversed the reduction in (P)RR protein abundance caused by miR-148a transfection in hepatic cells (**Fig 3B and 3C**). Antagonizing miR-148a using its inhibitors also effectively prevented miR-148a-induced reduction in LDL uptake (**Fig 3D and 3E**), likely due to recovered (P)RR and LDLR levels.

### Overexpressing (P)RR reversed MiR-148a-induced LDLR protein reduction

**Fig 4:**
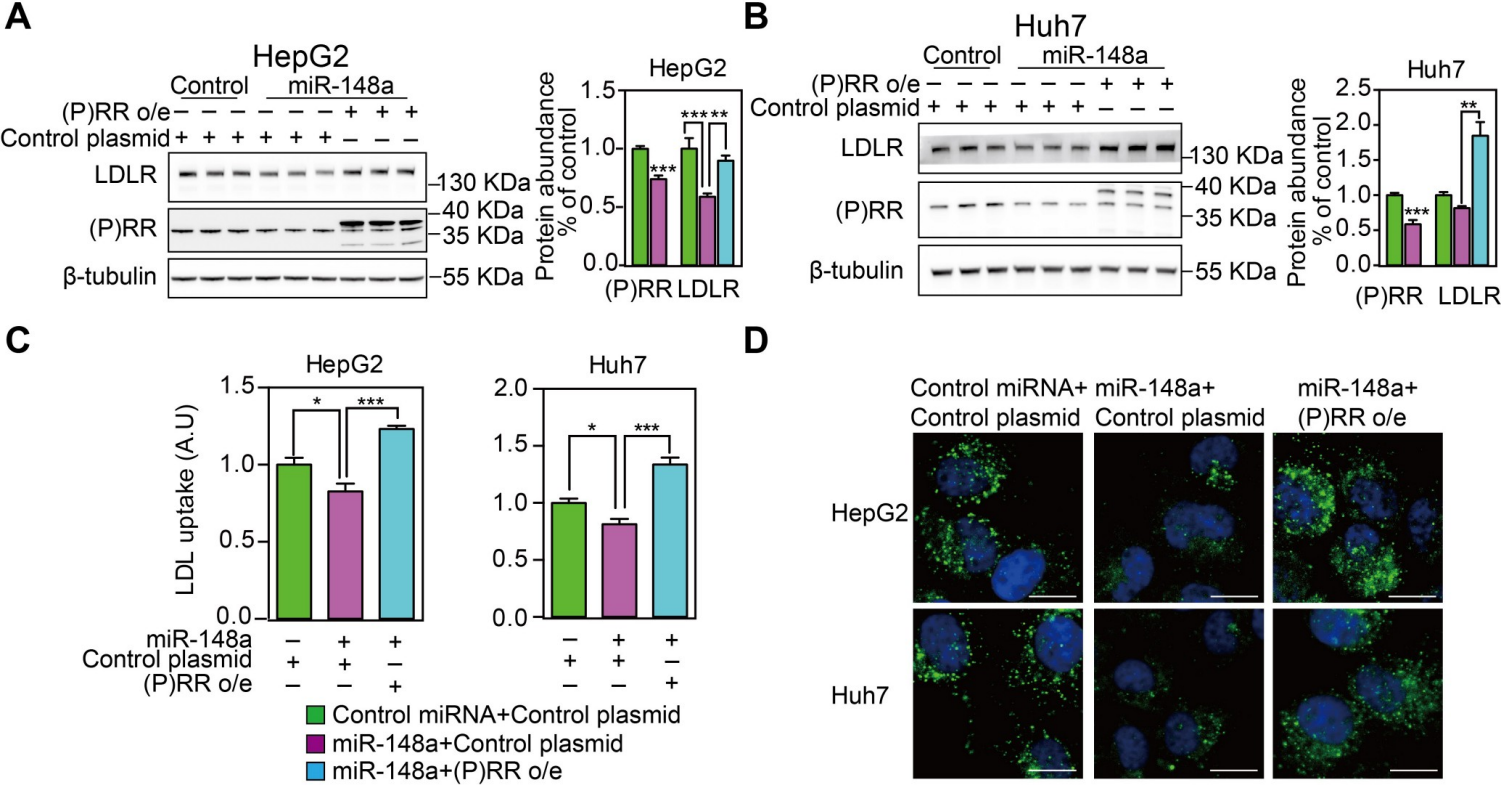
Overexpressing (P)RR antagonizes miR-148a-induced reduction in cellular LDL uptake and LDLR protein abundance. HepG2 and Huh7 cells were transfected with control miRNA or miR-148a together with control plasmid or (P)RR o/e plasmid. Cell were cultured under serum containing medium (**A&B**) or treated for 16h in LSM (**C&D**). **A & B**. Total lysates were blotted as indicated, and representative blot of 3 independent experiments in triplicates was shown. (P)RR and LDLR protein abundance were quantified and normalized to the level of tubulin in the same lysates, and expressed as ratio of control miRNA transfected. N = 9; *: p<0.05; **: p<0.01; ***: p<0.001. **C & D**. HepG2 and Huh7 cells were treated above, and subsequently incubated with 5 μg/mL Dylight488-labeled LDL for 3 hours. C. Representative fluorescence images. Scale bar, 10 μm. **D**. Quantitative measurement of LDL uptake. Results are from 3 independent experiments in triplicates. N = 9; *: p<0.01; **: p<0.001.

To further confirm if miR-148a regulates LDLR functions via (P)RR-LDLR axis, we overexpressed the (P)RR in hepatic cells in the presence of miR-148a. As expected, overexpressing the (P)RR reversed miR-148a-induced reduction in LDLR protein abundance in HepG2 and Huh7 cells (**Fig 4A and 4B**), and consequently cellular LDL uptake (**Fig 4C and 4D**). Collectively, these data show that miR-148a regulates LDL metabolism via (P)RR-LDLR axis.

## Discussion

In this study, we have identified miR-148a as a regulator for *(P)RR* expression in hepatic cells. MiR-148a is a member of the miR-148 family, which is consisted of miR-148a, miR-148b and miR-152, sharing similarities in nucleotide sequences. Interestingly, miR-152 has previously been shown to regulate *(P)RR* expression [19]. It is likely that this family plays an important role in regulating (P)RR functions. In viewing of this, we tested the effects of miR-148b on *(P)RR* expression and found miR-148b was capable of reducing (P)RR transcript levels and protein abundance, to a similar extent as miR-148a in hepatic cells (**S3 Fig**).

In the current study, we found that miR-148a reduces LDLR protein abundance and cellular LDL uptake. Our findings are in line with a previous report showing that miR-148a regulates LDL metabolism [27]. Yet, in contrast to the previous study, we found miR-148a did not affect *LDLR* transcript levels. It is unclear why such discrepancies exist, especially the same human hepatic cell lines were used in both studies. To rule out the possibility that the cells we used were contaminated with other cells, such as HeLa, we screened 19 short tandem repeat loci and the gender determining locus to determine their identity and confirmed that HepG2 and Huh7 cells used in the current study are correct cell lines (https://figshare.com/s/f460925897134ea7c6a3 and https://figshare.com/s/b01343989ab7727c0145). Furthermore, in this study, we found that reduction in LDLR protein abundance and LDL uptake induced by miR-148a were reversed by overexpressing the (P)RR, further arguing that (P)RR deficiency is the underlying cause for reduced cellular LDL uptake. Decreased (P)RR abundance will also reduce protein abundance of sortilin-1 (SORT1) [8]. SORT1 plays important roles in several cellular functions, such as neuronal survival and protein sorting [28, 29], and recently has been identified as a clearance receptor for LDL and an important determinant of circulating LDL levels [30–32]. In the current study, we found that miR-148a reduced SORT1 protein abundance but did not affect its transcript levels (**S4 Fig**), in line with our previous report showing that (P)RR regulates LDLR and SORT1 protein abundance post-transcriptionally [8]. These findings together favor that miR-148a regulates LDL metabolism via modulating the (P)RR, instead of directly regulating LDLR. However, it is still unclear why LDLR degradation accelerates in the absence of the (P)RR. But it is worthy to notice that membrane LDLR degradation rate is unaltered in the absence of the (P)RR[8]. Moreover, overexpressing SORT1 in the liver partially reversed the reduction in LDLR caused by (P)RR inhibition[16]. Additionally, inhibiting SORT1 also reduces protein abundance of LDLR in hepatic cell lines (HepG2 and Huh7) to a similar extent as inhibiting the (P)RR. Hence, it is possible that the (P)RR together with SORT1 are required for appropriate LDLR trafficking and absence of the (P)RR or SORT1 may cause mistargeting of newly synthesized LDLR to lysosome for degradation.

Recent studies reported that miR-148a levels were increased in the liver and adipose tissues of diet-induced obese mice, and positively associated with body-mass index in human [27, 33]. It is possible that elevated miR-148a levels under such conditions contribute to dysregulation of LDL metabolism, a common disorder in obese subjects. However, the role of the (P)RR in lipid metabolism is rather complicated. Inhibiting the (P)RR in the liver on one hand decreases LDL clearance but on the other hand also reduces hepatic VLDL secretion by limiting cholesterol and triglycerides synthesis in mice [16]. Moreover, inhibiting hepatic (P)RR also increases energy expenditure and thus attenuates diet-induced obesity in mice. It is thus possible that miR-148a could, by regulating (P)RR levels, play a role in regulating energy metabolism and lipid biosynthesis, in addition to regulating LDL metabolism.

Altered miR-148a levels have been observed under other pathophysiological conditions, such as in type 1 diabetes, gastrointestinal cancer, and ovarian cancer [22, 34–36]. It would be interesting to explore whether miR-148a could, by modulating (P)RR-mediated functions, such as regulating lipid metabolism, maintaining V-ATPase integrity, and activating tissue RAS, contribute to the onset and progression of such diseases. Among these diseases, it is interesting to notice that plasma miR-148a levels were elevated in LN patients and mice [37]. LN patients have higher risks for ischemic heart diseases [38, 39], which is likely caused by abnormalities in lipid metabolism. It is estimated that 30–50% of LN patients have high plasma total cholesterol and LDL levels [40–42]. It is unclear whether elevated miR-148a would contribute to hypercholesterolemia in LN patients by affecting LDL metabolism via the (P)RR. In fact, it is still a largely unsolved problem why patients with nephrotic syndrome (NS) develop hypercholesterolemia. Recent studies found that elevated hepatic IDOL and PCSK9 may contribute to NS-induced hypercholesterolemia [43], and ablation of hepatic PCSK9 attenuated NS-induced hypercholesterolemia in mice [44]. Yet, it is still unclear how renal dysfunctions in NS signals to the liver to regulate cholesterol metabolism. It is possible that miRNAs, including miR-148a, play an essential role in this process. Further studies are needed to clarify if miR-148a would contribute to hypercholesterolemia in NS.

In summary, we report that miR-148a reduces *(P)RR* expression by directly binding to the 3’-UTR of (P)RR messenger RNA in human hepatic cells. By suppressing *(P)RR* expression, miR-148a reduces LDLR protein abundance and consequently LDL metabolism. Our study highlights that miR-148a is a regulator for (P)RR expression and functions.

## Supporting information

S1 Fig Small interfering RNA targeting 3’-UTR regions of human LDLR effectively reduces luciferase activity. HEK293 cells were transfected with LDLR 3’-UTR luciferase reporter plasmid. Firefly luciferase activity was measured and corrected for Renilla luciferase activity in the same sample, and expressed as ratio of WT 3’-UTR transfected samples. Results are from four independent experiments in triplicates (N = 9). ***: p<0.01.
(TIF)

S2 Fig MiR-148a does not affect LDLR and ABCA1 expression under lipid-deficient condition. HepG2 cells were transfected with miR-148a for 32 hours, and culture medium were then changed to either LPDS-containing DMEM or LSM 16 hours prior to cell harvesting. (P)RR, LDLR and ABCA1 expression were analyzed. N = 3/group. ***: p< 0.001 (Two-tailed student T test).
(TIF)

S3 Fig MiR-148b regulates (P)RR expression by targeting its 3’-UTR sequences. **A**. An illustration showing that there are two predicted binding sites of miR-148b on the 3’-UTR region of human (P)RR. HepG2 and Huh7 cells were transfected with miR-148a or control miRNA for 48 hours, and gene expression and protein abundances were analyzed. **B.** *(P)RR* mRNA level was determined by quantitative PCR, and corrected for 36B4 level in the same sample and expressed as ratio of the control miRNA transfected. Results are from four independent experiments in triplicates. ***: p<0.001. **C**. Total cell lysates were blotted as indicated and a representative blot of 3 independent experiments in triplicates was shown. **D**. (P)RR protein abundance was quantified and normalized to the level of tubulin in the same lysates, and expressed as the relative ratio of (P)RR abundance in control miRNA transfected. N = 9; ***: P<0.001. **E.** An illustration showing two constructs (Mut1 and Mut2) which are mutated for the binding site for miR-148b on the 3’-UTR of human (P)RR, comparing to wildtype (WT) sequence. **F.** HEK293T cells were transfected with luciferase reporter plasmids constructed using wildtype (WT) and mutated (Mut1 and Mut2) 3’-UTR of human (P)RR, together with either control miRNA or miR-148b. Firefly luciferase activity was measured and corrected for Renilla luciferase activity in the same sample, and expressed as ratio of WT reporter plasmid transfected samples. Results are from four independent experiments in triplicates (N = 12). ##: WT+miR-148b vs. WT+control miRNA, p<0.01; N.S (not significant): Mut1+miR-148b vs. WT+miR-148b; &&: Mut2+miR-148b vs. WT, p<0.01.
(TIF)

S4 Fig MiR-148a reduces SORT1 protein abundance but does not affect its transcript levels. HepG2 and Huh7 cells were transfected with control miRNA or miR-148a for 48 hours, and protein abundance were analyzed. **A.** Total cell lysates were blotted as indicated and a representative blot of 3 independent experiments in triplicates was shown. SORT1 protein abundance was quantified and normalized to the level of tubulin in the same lysates, and expressed as the relative ratio of SORT1 abundance in control miRNA transfected. N = 9; **: p<0.01; ***: p<0.001. **B**. SORT1 mRNA level was determined by quantitative PCR, and corrected for 36B4 lvels in the same sample and expressed as ratio of the control miRNA transfected. Results are from three independent experiments performed in triplicates. N = 9. Anti-SORT1 (BD bioscience) was used at 1:1000.
(TIF)

S5 Fig (PDF)

S1 Table List of miRNA, siRNA and primers used in the current study. (DOCX)
