# Supplementary figures and images for: MicroRNA-148a regulates low-density lipoprotein metabolism by repressing the (pro)renin receptor
Source: PLoS One. 2020 May 21;15(5):e0225356. doi: 10.1371/journal.pone.0225356 (PMC7241754; doi:10.1371/journal.pone.0225356)

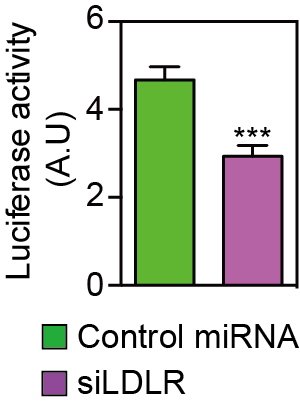

Supplement: S1 Fig — HEK293 cells were transfected with LDLR 3’-UTR luciferase reporter plasmid. Firefly luciferase activity was measured and corrected for Renilla luciferase activity in the same sample, and expressed as ratio of WT 3’-UTR transfected samples. Results are from four independent experiments in triplicates (N = 9). ***: p<0.01. (TIF) [file pone.0225356.s001.tif]

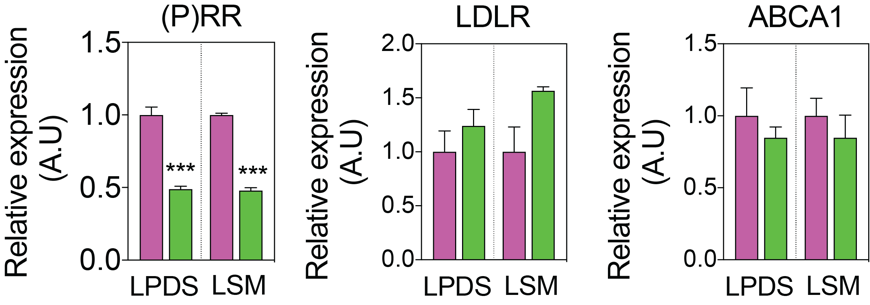

Supplement: S2 Fig — HepG2 cells were transfected with miR-148a for 32 hours, and culture medium were then changed to either LPDS-containing DMEM or LSM 16 hours prior to cell harvesting. (P)RR, LDLR and ABCA1 expression were analyzed. N = 3/group. ***: p< 0.001 (Two-tailed student T test). (TIF) [file pone.0225356.s002.tif]

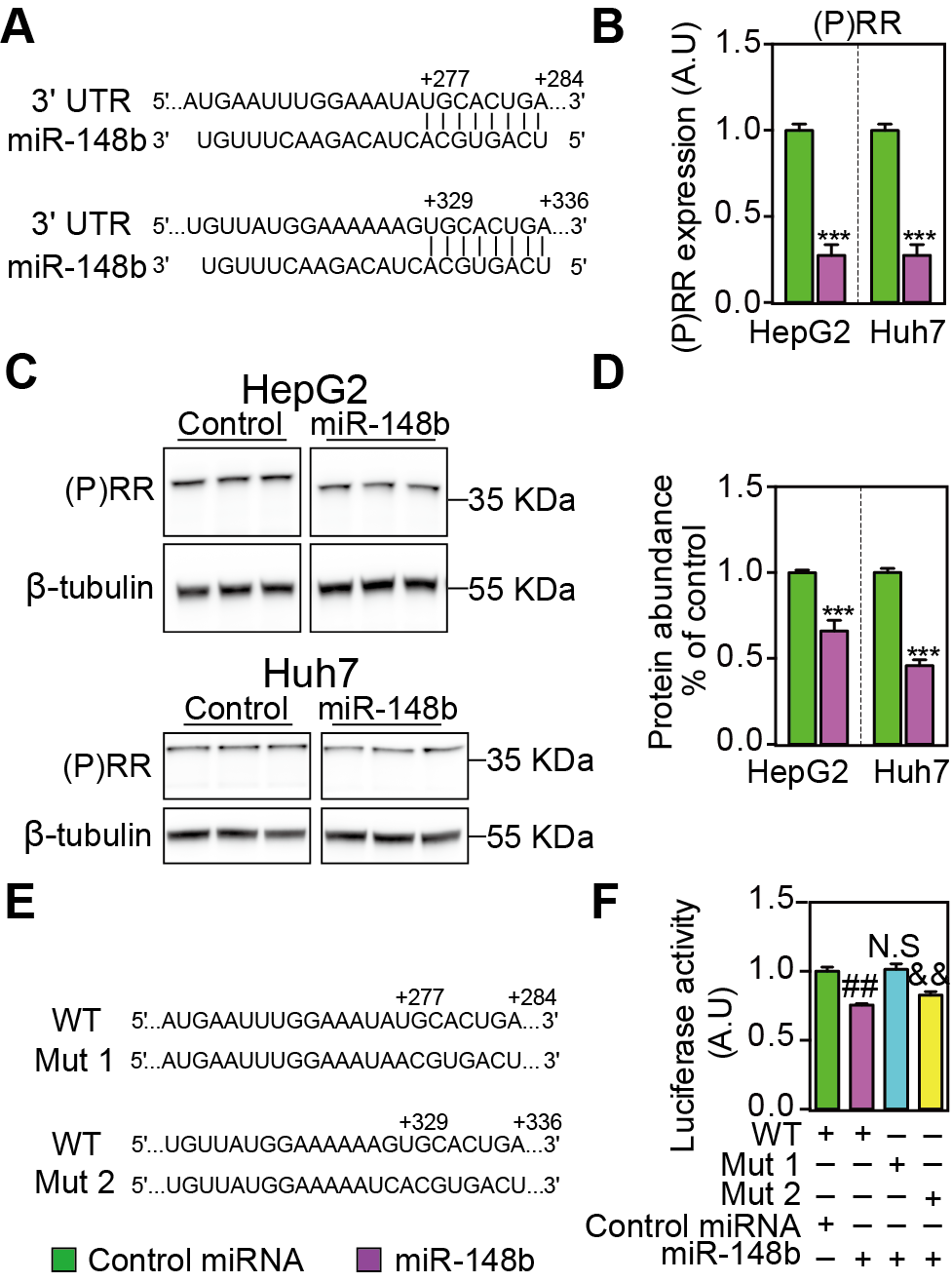

Supplement: S3 Fig — A. An illustration showing that there are two predicted binding sites of miR-148b on the 3’-UTR region of human (P)RR. HepG2 and Huh7 cells were transfected with miR-148a or control miRNA for 48 hours, and gene expression and protein abundances were analyzed. B. (P)RR mRNA level was determined by quantitative PCR, and corrected for 36B4 level in the same sample and expressed as ratio of the control miRNA transfected. Results are from four independent experiments in triplicates. ***: p<0.001. C. Total cell lysates were blotted as indicated and a representative blot of 3 independent experiments in triplicates was shown. D. (P)RR protein abundance was quantified and normalized to the level of tubulin in the same lysates, and expressed as the relative ratio of (P)RR abundance in control miRNA transfected. N = 9; ***: P<0.001. E. An illustration showing two constructs (Mut1 and Mut2) which are mutated for the binding site for miR-148b on the 3’-UTR of human (P)RR, comparing to wildtype (WT) sequence. F. HEK293T cells were transfected with luciferase reporter plasmids constructed using wildtype (WT) and mutated (Mut1 and Mut2) 3’-UTR of human (P)RR, together with either control miRNA or miR-148b. Firefly luciferase activity was measured and corrected for Renilla luciferase activity in the same sample, and expressed as ratio of WT reporter plasmid transfected samples. Results are from four independent experiments in triplicates (N = 12). ##: WT+miR-148b vs. WT+control miRNA, p<0.01; N.S (not significant): Mut1+miR-148b vs. WT+miR-148b; &&: Mut2+miR-148b vs. WT, p<0.01. (TIF) [file pone.0225356.s003.tif]

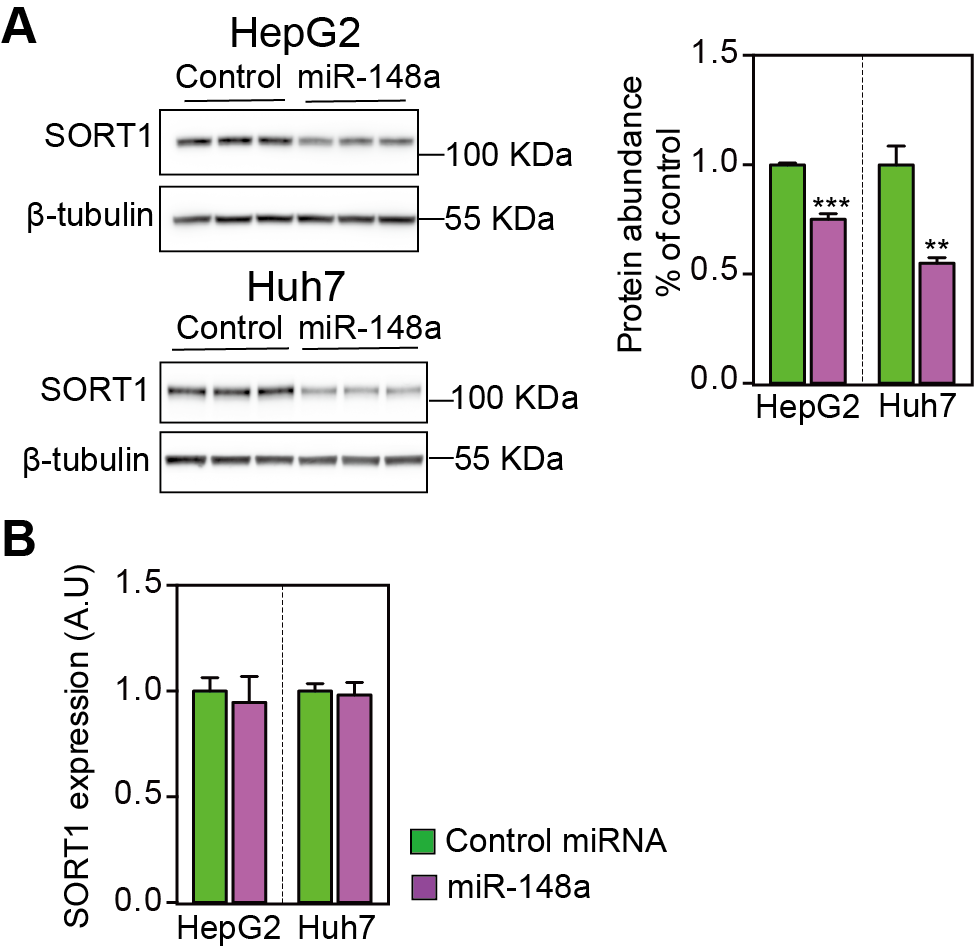

Supplement: S4 Fig — HepG2 and Huh7 cells were transfected with control miRNA or miR-148a for 48 hours, and protein abundance were analyzed. A. Total cell lysates were blotted as indicated and a representative blot of 3 independent experiments in triplicates was shown. SORT1 protein abundance was quantified and normalized to the level of tubulin in the same lysates, and expressed as the relative ratio of SORT1 abundance in control miRNA transfected. N = 9; **: p<0.01; ***: p<0.001. B. SORT1 mRNA level was determined by quantitative PCR, and corrected for 36B4 lvels in the same sample and expressed as ratio of the control miRNA transfected. Results are from three independent experiments performed in triplicates. N = 9. Anti-SORT1 (BD bioscience) was used at 1:1000. (TIF) [file pone.0225356.s004.tif]
