# Supplementary material for: MicroRNA-148a regulates low-density lipoprotein metabolism by repressing the (pro)renin receptor
Source: PLoS One. 2020 May 21;15(5):e0225356. doi: 10.1371/journal.pone.0225356 (PMC7241754; doi:10.1371/journal.pone.0225356)
Supplement: S5 Fig — (PDF) [file pone.0225356.s005.pdf]

F1_C_HepG2_β-tubulin

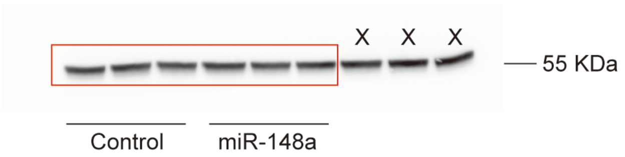

F1_C_HepG2_(P)RR

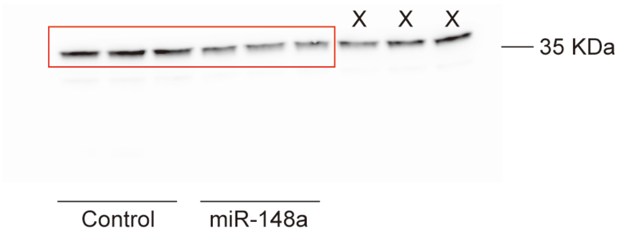

F1_C_Huh7_β-tubulin

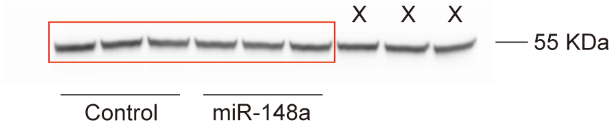

F1_C_Huh7_(P)RR

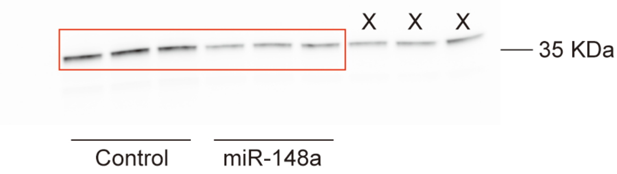

F2_E_HepG2_β-tubulin

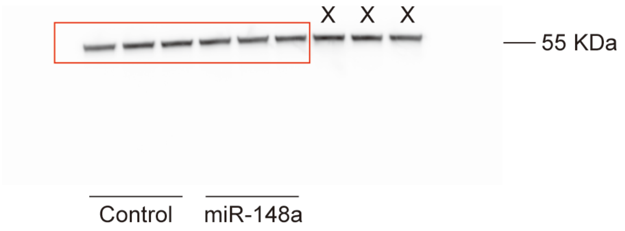

F2_E_HepG2_LDLR

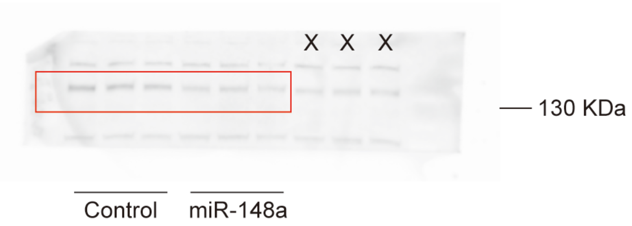

F2_E_Huh7_β-tubulin

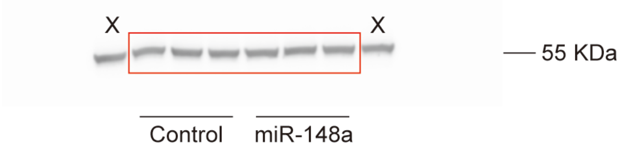

F1_E_Huh7_LDLR

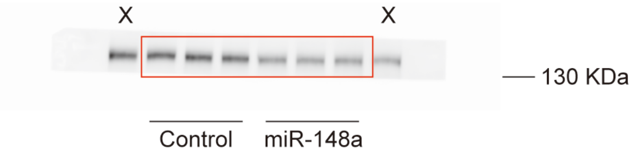

F3_B_HepG2_β-tubulin

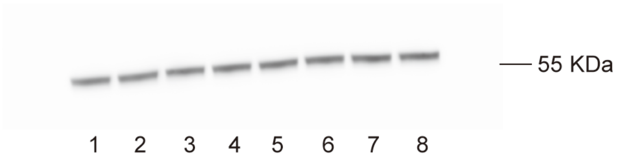

Lane 1 and 2: Control
Lane 3 and 4: miR-148a
Lane 5 and 6: miR-148a+Control inhibitor
Lane 7 and 8: miR-148a+miR-148a inhibitor

F3_B_HepG2_(P)RR

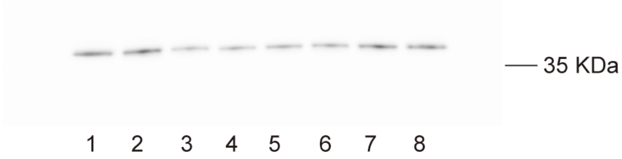

Lane 1 and 2: Control
Lane 3 and 4: miR-148a
Lane 5 and 6: miR-148a+Control inhibitor
Lane 7 and 8: miR-148a+miR-148a inhibitor

F3_B_HepG2_LDLR

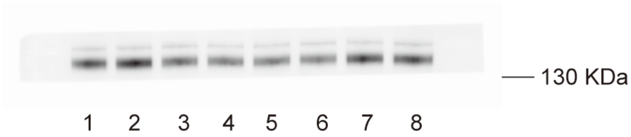

Lane 1 and 2: Control
Lane 3 and 4: miR-148a
Lane 5 and 6: miR-148a+Control inhibitor
Lane 7 and 8: miR-148a+miR-148a inhibitor

F3_C_Huh7_β-tubulin

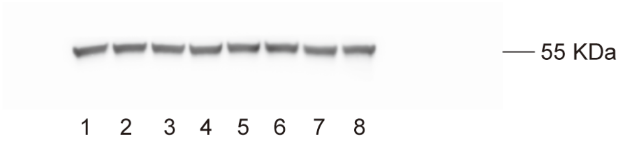

Lane 1 and 2: Control
Lane 3 and 4: miR-148a
Lane 5 and 6: miR-148a+Control inhibitor
Lane 7 and 8: miR-148a+miR-148a inhibitor

F3_C_Huh7_(P)RR

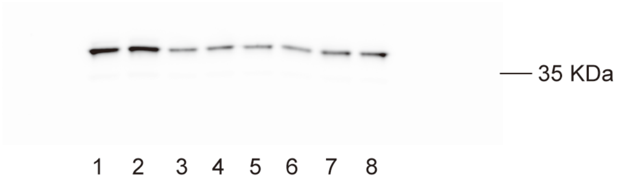

Lane 1 and 2: Control
Lane 3 and 4: miR-148a
Lane 5 and 6: miR-148a+Control inhibitor
Lane 7 and 8: miR-148a+miR-148a inhibitor

F3_C_Huh7_LDLR

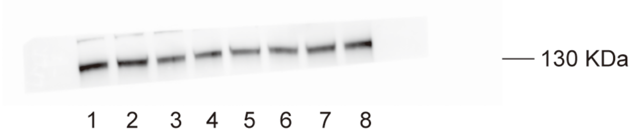

Lane 1 and 2: Control
Lane 3 and 4: miR-148a
Lane 5 and 6: miR-148a+Control inhibitor
Lane 7 and 8: miR-148a+miR-148a inhibitor

F4_A_HepG2_β-tubulin

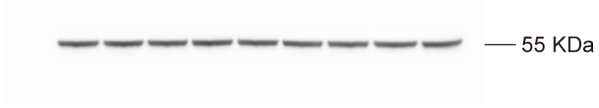

1 2 3 4 5 6 7 8 9

Lane 1,2,3: Control+Control plasmid
Lane 4,5,6: miR-148a+Control plasmid
Lane 7,8,9: miR-148a+(P)RR plasmid

F4_A_HepG2_(P)RR

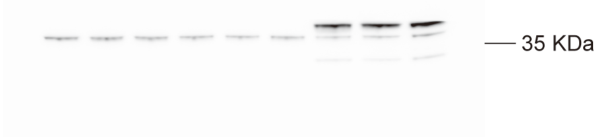

1 2 3 4 5 6 7 8 9

Lane 1,2,3: Control+Control plasmid
Lane 4,5,6: miR-148a+Control plasmid
Lane 7,8,9: miR-148a+(P)RR plasmid

F4_A_HepG2_LDLR

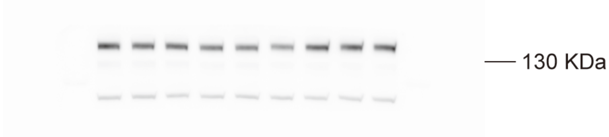

1 2 3 4 5 6 7 8 9

Lane 1,2,3: Control+Control plasmid
Lane 4,5,6: miR-148a+Control plasmid
Lane 7,8,9: miR-148a+(P)RR plasmid

F4_B_Huh7_β-tubulin

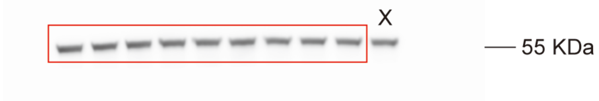

1 2 3 4 5 6 7 8 9

Lane 1,2,3: Control+Control plasmid
Lane 4,5,6: miR-148a+Control plasmid
Lane 7,8,9: miR-148a+(P)RR plasmid

F4_B_Huh7_(P)RR

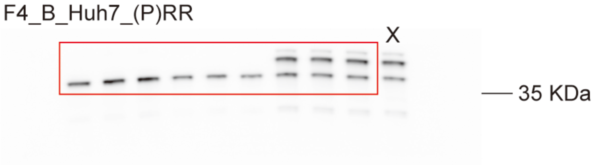

1 2 3 4 5 6 7 8 9

Lane 1,2,3: Control+Control plasmid
Lane 4,5,6: miR-148a+Control plasmid
Lane 7,8,9: miR-148a+(P)RR plasmid

F4_B_Huh7_LDLR

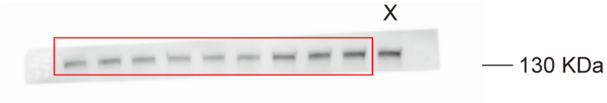

1 2 3 4 5 6 7 8 9

Lane 1,2,3: Control+Control plasmid
Lane 4,5,6: miR-148a+Control plasmid
Lane 7,8,9: miR-148a+(P)RR plasmid

S2_C_HepG2_β-tubulin

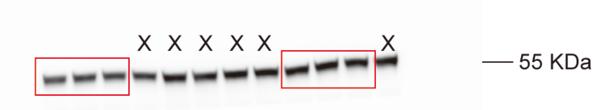

Control miR-148b

S2_C_HepG2_(P)RR

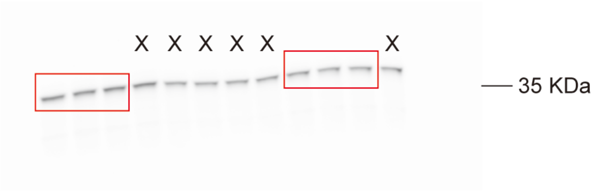

Control miR-148b

S2_C_Huh7_β-tubulin

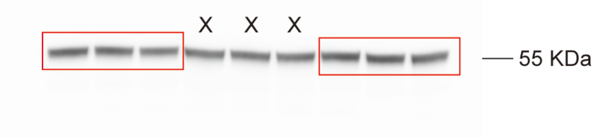

Control miR-148b

S2_C_Huh7_(P)RR

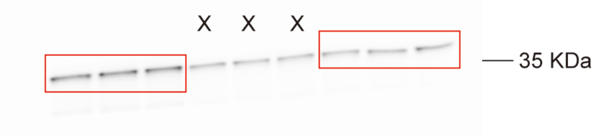

Control miR-148b

S3_A_HepG2_β-tubulin

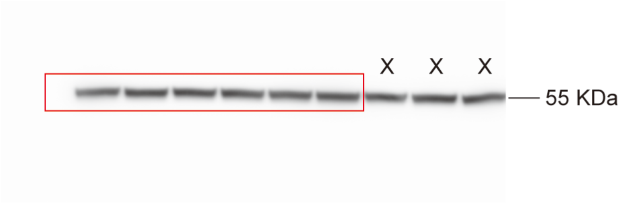

Control miR-148a

S3_A_HepG2_SORT1

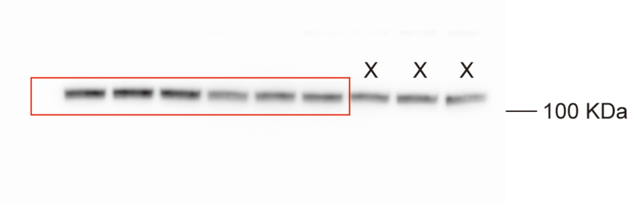

Control miR-148a

S3_A_Huh7_β-tubulin

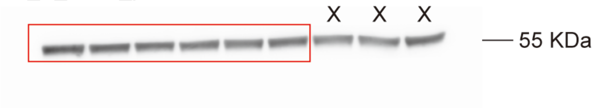

Control miR-148a

S3_A_Huh7_SORT1

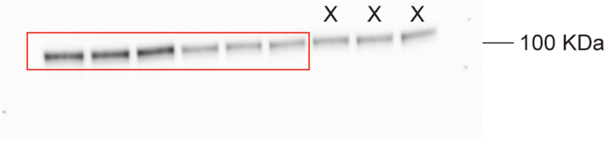

Control miR-148a
