# Supplementary material for: MicroRNA-148a regulates low-density lipoprotein metabolism by repressing the (pro)renin receptor
Source: PLoS One. 2020 May 21;15(5):e0225356. doi: 10.1371/journal.pone.0225356 (PMC7241754; doi:10.1371/journal.pone.0225356)
Supplement: S1 Table — (DOCX) [file pone.0225356.s006.docx]

**S1 Table. List of miRNA, siRNA and primers used in the current study.**

| miRNA/siRNA/primer | sequence |
| --- | --- |
| Control miRNA | 5'-UUCUCCGAACGUGUCACGUTT-3' |
|  | 5'-ACGUGACACGUUCGGAGAATT-3’ |
| miR-148a | 5'-UCAGUGCACUACAGAACUUUGU-3' |
|  | 5'-AAAGUUCUGUAGUGCACUGAUU-3' |
| miR-148b | 5'-UCAGUGCAUCACAGAACUUUGU-3' |
|  | 5'-AAAGUUCUGUGAUGCACUGAUU-3' |
| Control inhibitor | 5’-CAGUACUUUUGUGUAGUACAA-3’ |
| miR-148a inhibitor | 5'-ACAAAGUUCUGUAGUGCACUGA-3' |
| (P)RR | (Forward) 5'-CTGAACTGCAAGTGCTACATGA-3' |
|  | (Reverse) 5'-AACCTGCCAGCTCCAGTG-3' |
| LDLR | (Forward) 5'-CTACAAGTGGGTCTGCGATG-3' |
|  | (Reverse) 5'-TTTGCAGGTGACAGACAAGC-3' |
| ABCA1 | (Forward) 5’-CCACAAAAACATTGCTGCAT-3’ |
|  | (Reverse) 5’-GTCACTCCAGCTTCTCATGCT-3’ |
| SORT1 | (Forward) 5'-GGCATCATTGTGGCCATT-3' |
|  | (Reverse) 5'-TTGACCTTCGTCTGTGGAGA-3' |
| 36B4 | (Forward) 5'-TCTACAACCCTGAAGTGCTTGAT-3' |
|  | (Reverse) 5'-CAATCTGCAGACAGACACTGG-3' |
| siLDLR | 5'-CCUGAGUCACUGGUCACCCUUAAUA-3' |
|  | 5'-UAUUAAGGGUGACCAGUGACUCAGG-3' |
